# Cortical bone porosity is spatially heterogeneous and VEGF dependent in male bone

**DOI:** 10.1242/dmm.052609

**Published:** 2026-01-26

**Authors:** Jacob Trend, Jacob Keen, Aikta Sharma, Alisha Sharma, Lysanne Michels, Patricia Goggin, Philipp Schneider, Katrin Deinhardt, Claire E. Clarkin

**Affiliations:** ^1^ School of Biological Sciences, Faculty of Environmental and Life Sciences, University of Southampton, Southampton SO17 1BJ, UK; ^2^ Faculty of Engineering and Physical Sciences, University of Southampton, Southampton SO17 1BJ, UK; ^3^ Department of Mechanical Engineering, Faculty of Engineering Sciences, University College London, London WC1E 7JE, UK; ^4^ Biomedical Imaging Unit, University Hospital Southampton NHS Foundation Trust, Southampton SO16 6YD, UK; ^5^ Bioengineering Science Research Group, Faculty of Engineering and Physical Sciences, University of Southampton, Southampton SO17 1BJ, UK; ^6^ High-Performance Vision Systems, Center for Vision, Automation and Control, AIT Austrian Institute of Technology, Giefinggasse 4, 1210 Vienna, Austria; ^7^ Zellbiologie Universität Bremen FB02 Biologie/Chemie NW2, B2235, Universität Bremen, Leobener Str. 5, 28359 Bremen, Germany

**Keywords:** Bone, Osteocyte, Osteoblast, Vascular, Computed tomography, VEGF

## Abstract

Cortical bone is highly porous and composed of an interconnecting network of vascular canals and osteocyte lacunae. Our understanding of the mechanisms coupling vascular: lacunar spatial organisation in cortical bone is poorly understood. Defining cellular cross-talk mechanisms could be key in identification of reciprocal molecular signals driving increased cortical porosity with age. Driven by the hypothesis that porosity within bone is heterogeneous and influenced by region-specific spatial cues, we utilised synchrotron X-ray computed tomography to characterise intracortical canal and osteocyte lacunae distribution, morphology and spatial arrangements in healthy and pathological murine bone. We found that the posterior region of the tibiofibular junction (TFJ) exhibited the highest levels of cortical porosity and highest canal number density compared to other regions. The volume of osteocyte lacunae positioned proximal to cortical vascular canals was highest in the posterior region. Following deletion of bone-derived VEGF, the region-specific effects on lacunar: vascular arrangements described in the wild-type TFJ were lost. Our results describe spatial diversity in osteocyte lacunae size within the bone cortex, which associates with vascular canal arrangements maintained by VEGF.

## Teaser

**Summary:** This study uses synchrotron X-ray computed tomography to characterise intracortical canal and osteocyte lacunae distribution, morphology and spatial arrangements in healthy and pathological murine bone.

## INTRODUCTION

Degenerative bone diseases, including osteoporosis, are characterised by increased cortical porosity leading to bones that are more prone to fracture, with 76% of the reduction in bone strength in aged bone attributable to altered cortical porosity (McCalden et al., 1993), and the intracortical structure of bone at microscopic scales comprising vascular canals and osteocyte lacunae (Schneider et al., 2009, 2013).

Intracortical canals are structural components of the cortical bone that surround and protect the soft tissue of bone vasculature, which is required to sustain bone cell viability, growth and repair (Bonewald, 2011). Throughout life, the bone vasculature retains specialised structural and functional roles, with vascular heterogeneity existing at both the organ (Marcu et al., 2018) and cellular (Ramasamy et al., 2014, 2015; Ramasamy et al., 2016) level, suggesting that distinct phenotypes of the bone vasculature exist with specific functions. In bone, specific vascular endothelial subtypes have been described and linked to angiogenic potential driving osteogenesis (Ramasamy et al., 2015, 2016; Wang et al., 2017; Zhu et al., 2019). In addition to functional variability, heterogeneity within intracortical canal distribution within specific bone regions has been documented in murine (Núñez et al., 2018; Schneider et al., 2007) and human (Perilli et al., 2015) bone. However, how the spatial arrangements of intracortical canals within bone develop and impact bone cell behaviour, function and structural integrity remains unclear.

The cortical microarchitecture is organised on a number of hierarchical levels, which also include the smaller-scale lacunar–canalicular network (LCN), the main communication pathway between osteocytes providing a conduit for interstitial fluid, which is critical in the maintenance of bone homeostasis. The adequate supply of nutrients, disposal of waste products and exchange of regulatory signals through the LCN is essential for the viability and function of osteocytes contained within their lacunae (Bonewald, 2007; Kogianni and Noble, 2007). The LCN has been demonstrated to play a critical role in spatial bone density, and the location of osteocytes and the LCN within the mineralised bone cortex are thought to enable osteocyte function as both mechanosensory cells (Tatsumi et al., 2007; Fritton and Weinbaum, 2009) and regulators of bone (re)modelling (Bonewald, 2011), sensitive to stimuli including mechanical (Zhao et al., 2022) and lactation (Qing et al., 2012). Mechanical stress compresses the bone cortex, driving interstitial fluid flow through the LCN (Bonewald, 2011), and physical strains within the LCN are transduced by osteocytes into biochemical cues driving bone resorption by osteoclasts and bone deposition by osteoblasts (Bakker et al., 2001). Regional heterogeneity within the canalicular length, density and fluid flow is observed across murine bone (van Tol et al., 2020), suggestive that functional heterogeneity exists within the LCN in response to heterogeneous strain distribution.

We previously reported intracortical canal sensitivity to ageing, which was localised at the murine tibiofibular junction (TFJ) (Núñez et al., 2018). Specifically, we found that, in aged murine bone, reductions in intracortical canal density were localised specifically to the posterior region of the TFJ. Although other studies have compartmentalised (segmented into different regions, typically quadrants for long bones) the bone cortex for morphometric analysis (Núñez et al., 2018; van Tol et al., 2020; Uniyal et al., 2021; Schneider et al., 2007; Cole et al., 2022; Chiba et al., 2013), manual selection of regions can lead to inter- and intra-observer errors, and automated regionalisation of high-resolution computed tomography (CT) scans is likely to be more reliable.

Driven by the hypothesis that cortical porosity comprising intracortical canals and osteocyte lacunae within cortical bone is heterogeneous and influenced by region-specific spatial cues, we utilised synchrotron X-ray (SR) CT and developed a workflow to automate regional characterisation of intracortical canal and osteocyte lacunae distribution, morphology and spatial arrangements. Here, we (1) used an automated means to regionally segment SR CT scans of the bone cortex and associated porosity into anterior posterior, lateral and medial quadrants, (2) developed a 3D lacunar distance mapping technique to evaluate how osteocyte lacunae and the intracortical canals are spatially organised across the four quadrants, and (3) investigated how bone pathology driven through bone-specific deletion of vascular endothelial growth factor (VEGF) influences vascular: lacunar organisation.

## RESULTS

### Regional distribution of cortical porosity in the murine cortex

The TBJ (Fig. 3A) was scanned across wild-type (WT) male mice by SR CT with 3D rendering of intracortical canals (Fig. 3B) and osteocyte lacunae volumes (Fig. 3C) produced. Microstructural analysis showed that cortical porosity averaged across the entire TFJ presented as a percentage of total cortical volume (Ct.Po%) was 1.38% [±0.01% (s.d.); see Fig. 3], in line with previous observations (Willey et al., 2008) in WT mice of a similar age. Upon regionalisation, the posterior region (2.22±0.62%) exhibited highest %Ct.Po compared to that in the anterior (0.888±0.109%, *P*=0.005), lateral (1.142±0.076%, *P*=0.017) and medial (1.292±0.157%, *P*=0.036) regions (Fig. 3D).

**Fig. 1.**
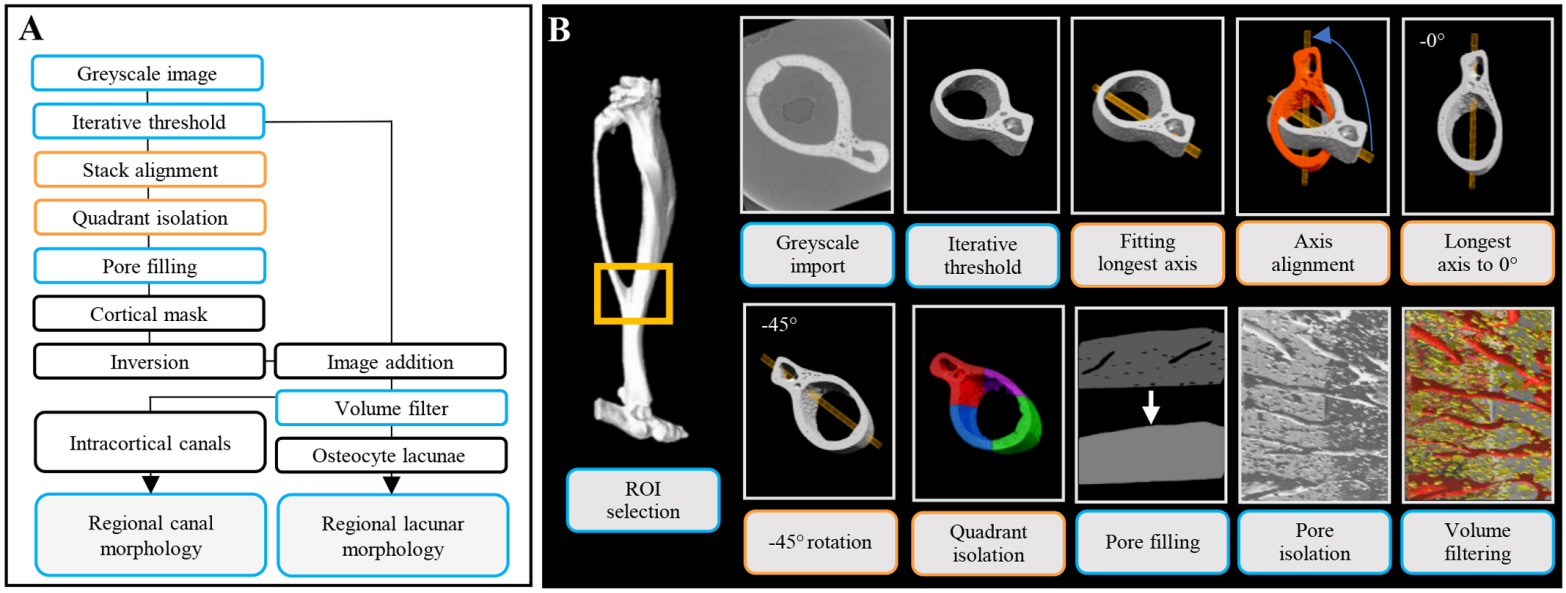
**Workflow for the automated regionalisation of the murine tibiofibular junction.** (A) Image processing workflow for automated regionalisation of the murine tibiofibular junction (TFJ) and the assessment of cortical porosity. (B) Synchrotron X-ray computed tomography (SR CT) scans of the murine TFJ (yellow box) were imported into Fiji. Image stacks were aligned by fitting an ellipsoid to the TFJ, with the TFJ's longest axis manipulated to 0°. The aligned TFJ was split into quadrants. Regional cortical porosity was extracted and subjected to a volume filter to discard noise (0-25 μm^3^) and to isolate osteocyte lacunae (25-2500 μm^3^) and intracortical canals (>2500 μm^3^). ROI, region of interest.

**Fig. 2.**
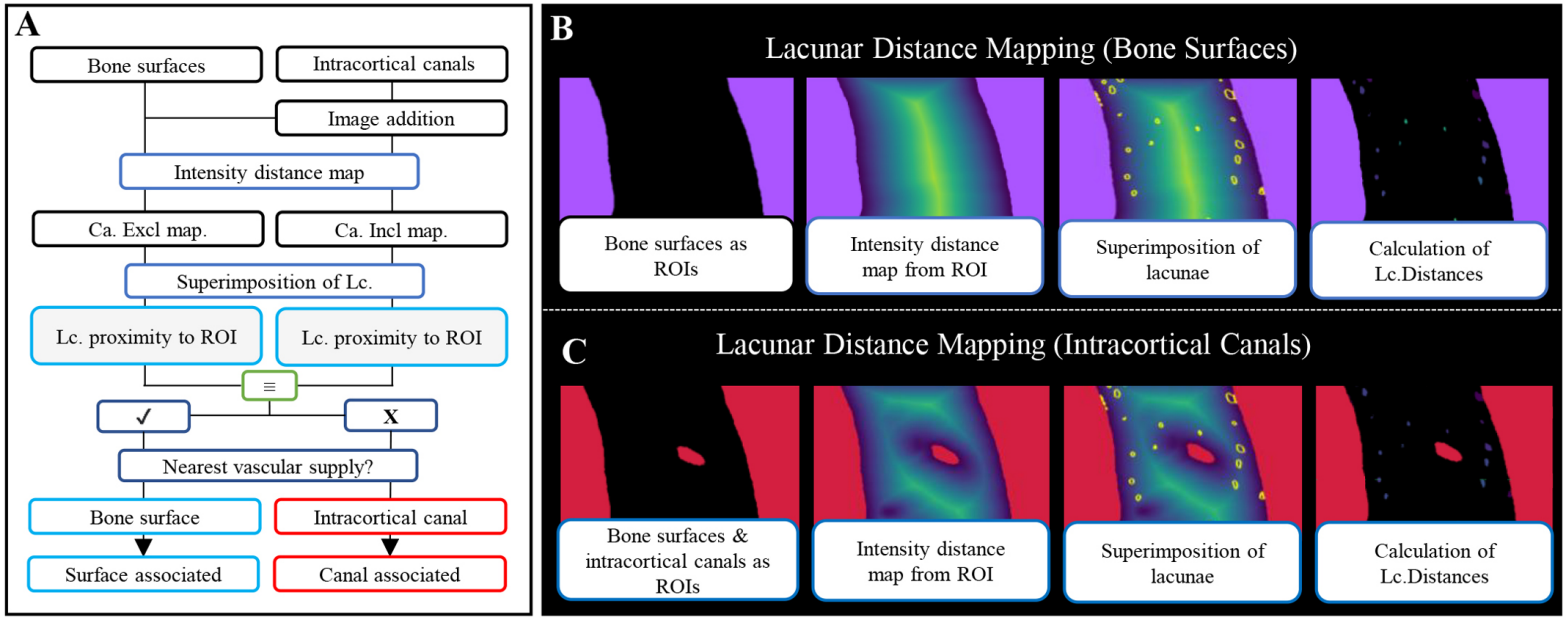
**Image processing workflow for lacunae distance mapping.** (A) Image processing workflow for lacunae distance mapping to intracortical and to vascular supply of cortical surfaces i.e. periosteal and endosteal. (B,C) A lacunar distance map was created from the inverted intracortical canal mask to map the distance of each pixel within the bone cortex to the nearest bone surface (B) or intracortical canal (C). Lacunae image stacks were imported into Dragonfly and converted to a multi-ROI, with each lacuna superimposed onto a distance map across the bone surface. Ca.Excl, canals excluded; Ca.Incl, canals included; Lc., lacunar.

**Fig. 3.**
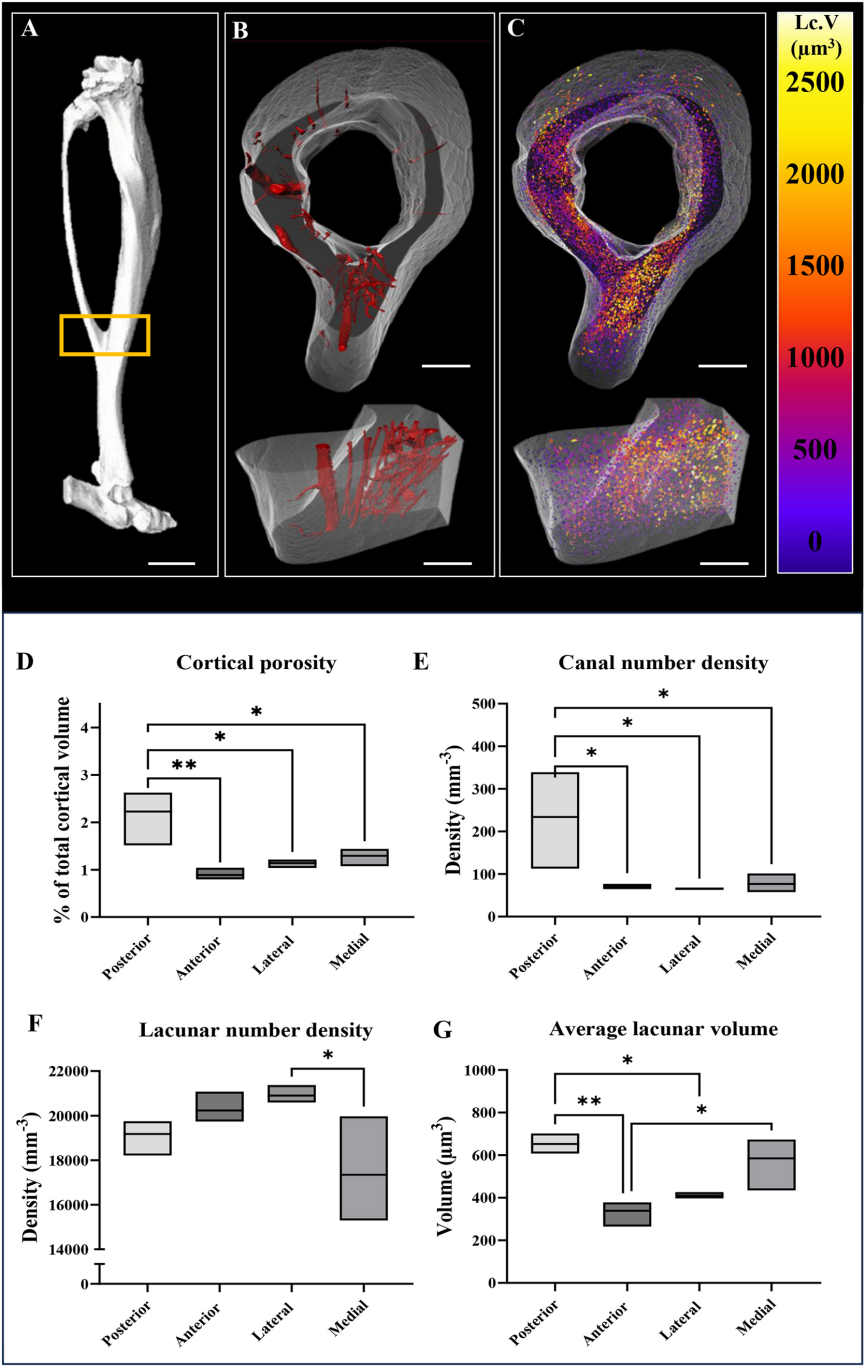
**Regionalisation of murine cortical bone reveals region-specific microstructural properties.** (A-C) Regional microstructure was quantified at the TFJ (yellow box) (A) and separated into intracortical canals (B) and osteocyte lacunae (C), mapped here by size with largest lacunae coloured in yellow and smallest lacunae coloured purple. (D-G) Cortical porosity (D), canal number density (E), lacunar number density (F) and average lacunar volume (G) were regionalised and quantified. Scale bars: 200 μm. Data shown as mean±s.d. and assessed by one-way ANOVA, *n*=3 bones. *P<0.05, **P<0.01.

Canal number density (Fig. 3E) was highest in the posterior region compared to that in the anterior (233.99±92.99 mm^−3^ versus 71.72±5.11 mm^−3^, *P*=0.042), lateral (65.18±1.99 mm^−3^, *P*=0.030) and medial (76.85±18.14 mm^−3^, *P*=0.037) regions.

Lacunar number density (Fig. 3F) was higher in the lateral region than in the medial region (20,901.41±337.79 mm^−3^ versus 17,345.26±1950.36 mm^−3^, *P*=0.046) (Fig. 3F). For average lacunar volume (Lc.V) (Fig. 3G), lacunae were largest in the posterior region compared to those in the anterior (652.74±38.39 µm^3^ versus 338.62±52.28 µm^3^, *P*=0.004) and lateral (410.02±12.0016 µm^3^, *P*=0.019) regions, while medial lacunae were also larger than anterior lacunae (584.78±106.43 µm^3^, *P*=0.180). Lc.V/cortical total bone volume (Ct.TV) was also higher in the posterior and medial regions than in the anterior region [1.25±0.13% (*P*=0.003), 1.18±0.22% (*P*=0.007) versus 0.59±0.16%, respectively].

### 3D lacunar distance mapping of osteocyte lacunae to intracortical canals and bone surfaces (endosteal and periosteal)

**Fig. 4.**
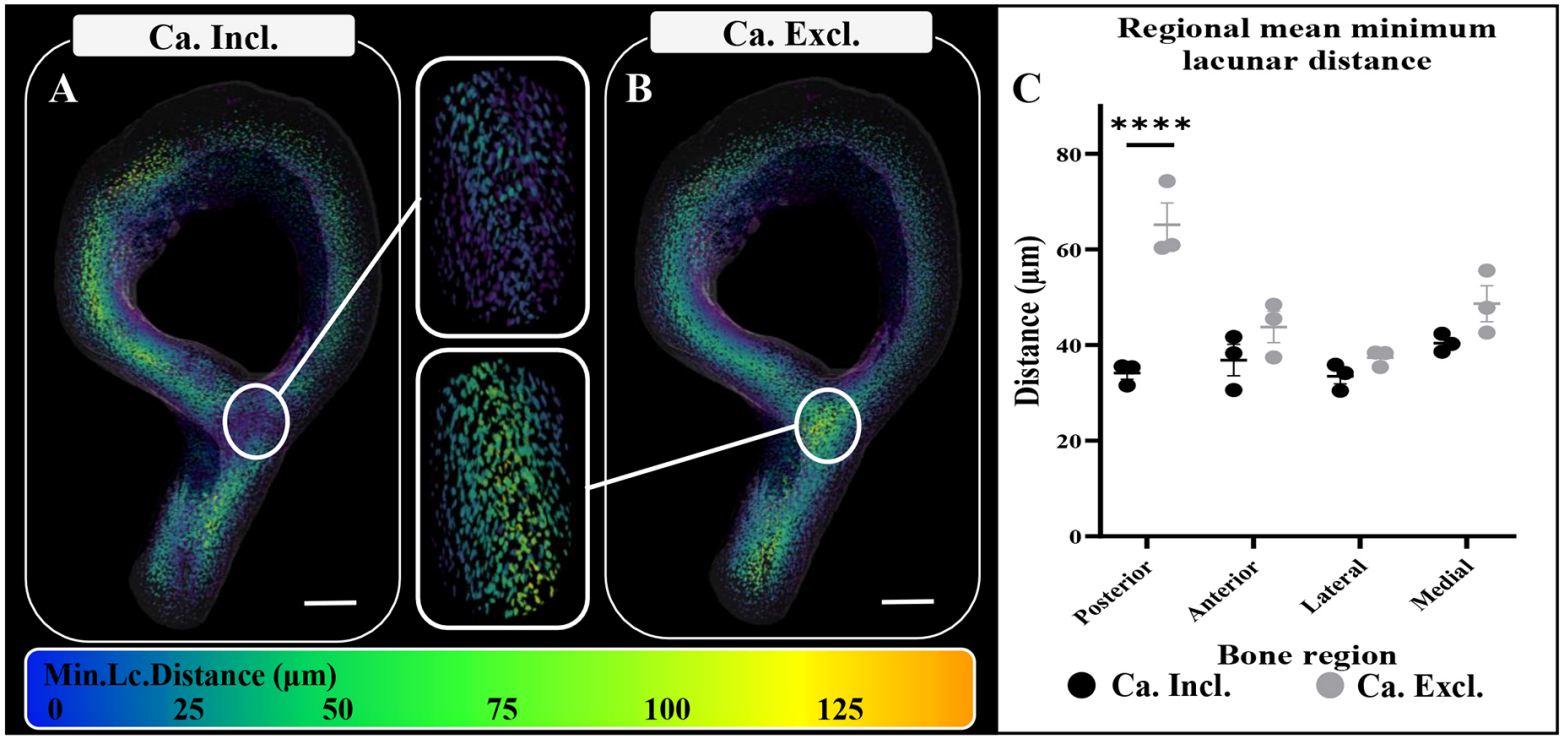
**3D lacunar distance mapping of osteocyte lacunae to intracortical canals and bone surfaces.** (A-C) 3D spatial mapping of mean minimum lacunar distance (Mm.Lc.Distance) from both intracortical canals and bone surfaces, with canals included (Ca.Incl; A) and with canals excluded (Ca.Excl; B) from the analysis, and then regionalised (C). Scale bars: 200 μm. Data shown as mean±s.d. and assessed by two-way ANOVA, *n*=3 bones. ****P<0.0001.

3D distance mapping was used to assess the 3D spatial organisation of osteocyte lacunae and the vascular supplies of the cortical bone, which contains endosteal/periosteal surfaces and intracortical canals within each cortical region. Whole cross-sectional analysis of the TFJ revealed that the mean minimum lacunar distance (Mm.Lc.D) to vascular supply was 36.2±0.99 µm, and regionalisation revealed no significant difference in Mm.Lc.D between anterior, posterior, lateral and medial quadrants. To investigate differences in the association of osteocytes with different vascular sources, i.e. intracortical canals and bone surfaces, distance maps were computed in two ways: in relation to both endosteal/periosteal surfaces and intracortical canals [canals included (Ca.Incl) distance maps; Fig. 4A] and also solely in relation to bone surfaces [canals excluded (Ca.Excl) distance maps; Fig. 4B]. Whole cross-sectional analysis of lacunar volume in Ca.Incl distance maps compared with Ca.Excl maps showed that removal of intracortical canals increased Mm.Lc.D to vascular supply from 36.23 µm (±0.9 µm, *P*=0.002) to 48.8 µm (±1.9 µm) across the TFJ. Regionalisation revealed that when canals are excluded from the posterior region, the distance of lacunae to bone surface was higher than in all other regions [to 34.2 µm (±1.8 µm) Mm.Lc.D from 60.9 µm (±6.4 µm) (*P*<0.0001)]. For the anterior (*P*=0.346), lateral (*P*=0.81) and medial (*P*=0.19) regions, no significant difference in distance to bone surfaces was detected (Fig. 4C) when intracortical canals were removed. Further analyses indicated that, within the posterior region, with 26.8% (±2.9%) of lacunae present >100 μm from a bone surface and in which intracortical canals were excluded, Ca.Incl greatly reduced this proportion (3.4±0.6%, *P*<0.0001; data not shown), suggesting that the lacunae in the posterior region are more heavily reliant on the intracortical canals for oxygen and nutrient transport and survival than those in the other regions.

### Canal-associated lacunae are largest in the posterior region

**Fig. 5.**
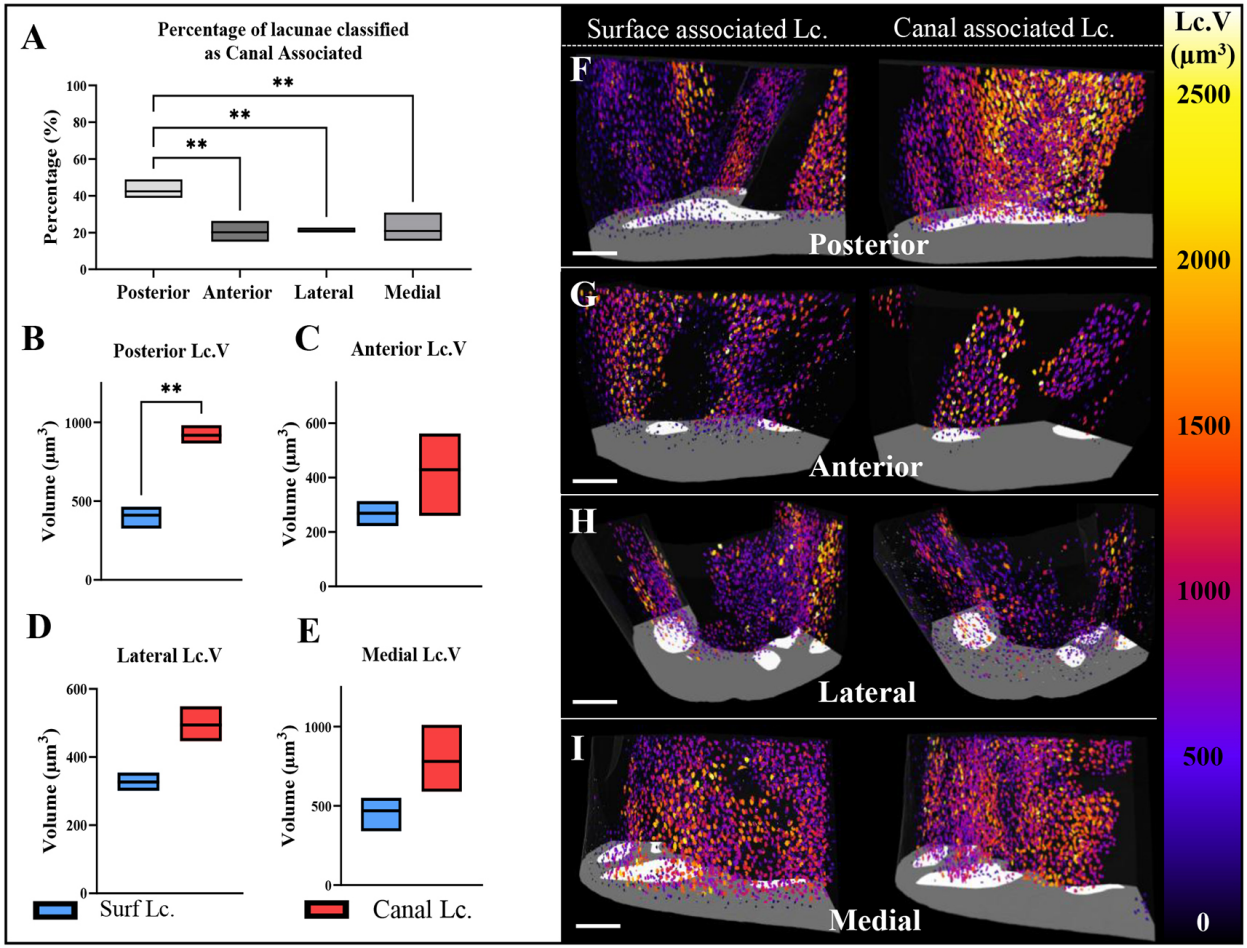
**Lacunae associated with intracortical canals are larger than surface lacunae in the posterior region.** (A) The proportion of lacunae defined as canal associated within each region. (B-E) The mean volume of canal- and surface-associated lacunae was calculated in the posterior (B), anterior (C), lateral (D) and medial (E) regions. (F-I) 3D visualisation of canal- and surface-associated osteocyte lacunae volume distributions in the posterior (F), anterior (G), lateral (H) and medial regions (I). Larger lacunae are coloured in orange/yellow, and smaller lacunae are coloured in blue/purple. Lc.V, lacunar volume. Scale bars: 200μm. Data shown as mean±s.d. and assessed by a one-way ANOVA with subsequent post hoc analysis, *n*=3 bones. **P<0.01.

To determine whether the heterogeneity of intracortical canals identified in the posterior TFJ influenced the heterogeneity of osteocyte lacunae volume in this region, lacunar populations were separated into canal-associated and surface-associated lacunae. Most (42.44±4.55%) lacunae classified as canal associated were localised to the posterior region, and the numbers were significantly higher than those in the anterior (20.11±4.602%, *P*=0.007), lateral (21.02±1.18%, *P*=0.009) and medial (20.86±7.04%, *P*=0.009) quadrants (Fig. 5A).

In the posterior region, canal-associated lacunae (919±48 µm^3^) were larger than surface-associated lacunae (411±60 µm^3^, *P*<0.001) (Fig. 5B). In the anterior (Fig. 5C), lateral (Fig. 5D) and medial (Fig. 5E) regions, no significant difference in the average lacunar volume of canal-associated and surface-associated lacunae was found. Further, posterior canal-associated lacunae (919±48 µm^3^) were larger than those in the anterior (429±126 µm^3^, *P*=0.010) and lateral (494±42 µm^3^, *P*=0.022) regions. 3D reconstructions highlighting the larger canal-associated lacunae evident in the posterior region than those in the anterior, lateral and medial compartments are shown in Fig. 5F-I.

### Effects of VEGF deletion on vascular: lacunar spatial arrangements

**Fig. 6.**
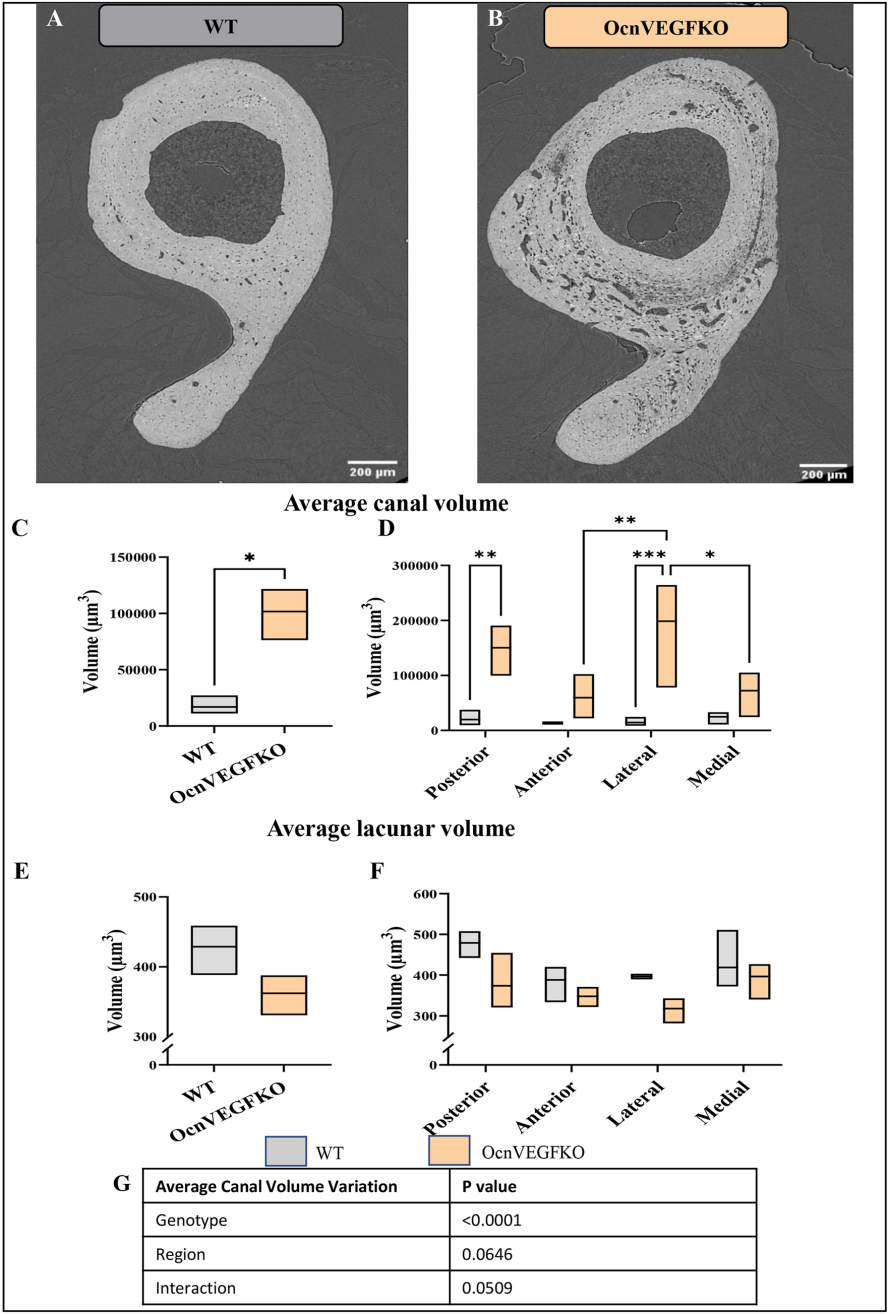
**Conditional VEGF deletion disrupts intracortical canal volume in a region-specific manner.** (A,B) High-resolution SR CT visualises male WT (A) and osteocalcin-specific VEGF knockout (OcnVEGFKO) (B) cortical bone. (C,D) Average canal volume of the whole TFJ (C) and regionalised (D) in WT and OcnVEGFKO bones. (E,F) Average lacunar volume of the whole TFJ (E) and regionalised (F) in WT and OcnVEGFKO bones. (G) A Tukey's comparison was carried to test interaction between genotype, region and interaction. Scale bars: 200 μm. Data shown as mean±s.d. and assessed by two-way ANOVA, *n*=3 bones. *P<0.05, **P<0.01, ***P<0.001.

To establish whether differences in vascular canal number density and canal-associated lacunae volume observed in the posterior region are accompanied by regional differences in growth factor secretion within regions, we undertook the analysis above in TFJs of adult male osteocalcin-specific VEGF knockout (OcnVEGFKO) mice (Goring et al., 2019; Sharma et al., 2021). We have previously shown that disruption of VEGF in osteocalcin-expressing cells is sexually dimorphic and, in male mice, leads to a highly vascularised TFJ (Goring et al., 2019; Fig. 6A,B). Here, male OcnVEGFKO cortices were highly porous, with canal volume significantly higher across TFJ cross-sections compared with TFJ cross-sections of male WT cortices (Fig. 6A-C). Conditional VEGF deletion also disrupts intracortical canal volume in a region-specific manner (Fig. 6D). The anterior and medial regions exhibited a trend towards lower canal volume than that observed in the posterior and lateral quadrants, although this trend did not reach significance. No significant difference in average lacunar volume was present across the entire cross-section of the TFJ (Fig. 6E) or when regionalised (Fig. 6F). A Tukey's comparison was carried out, showing no significant interaction between region and interaction (Fig. 6G).

**Fig. 7.**
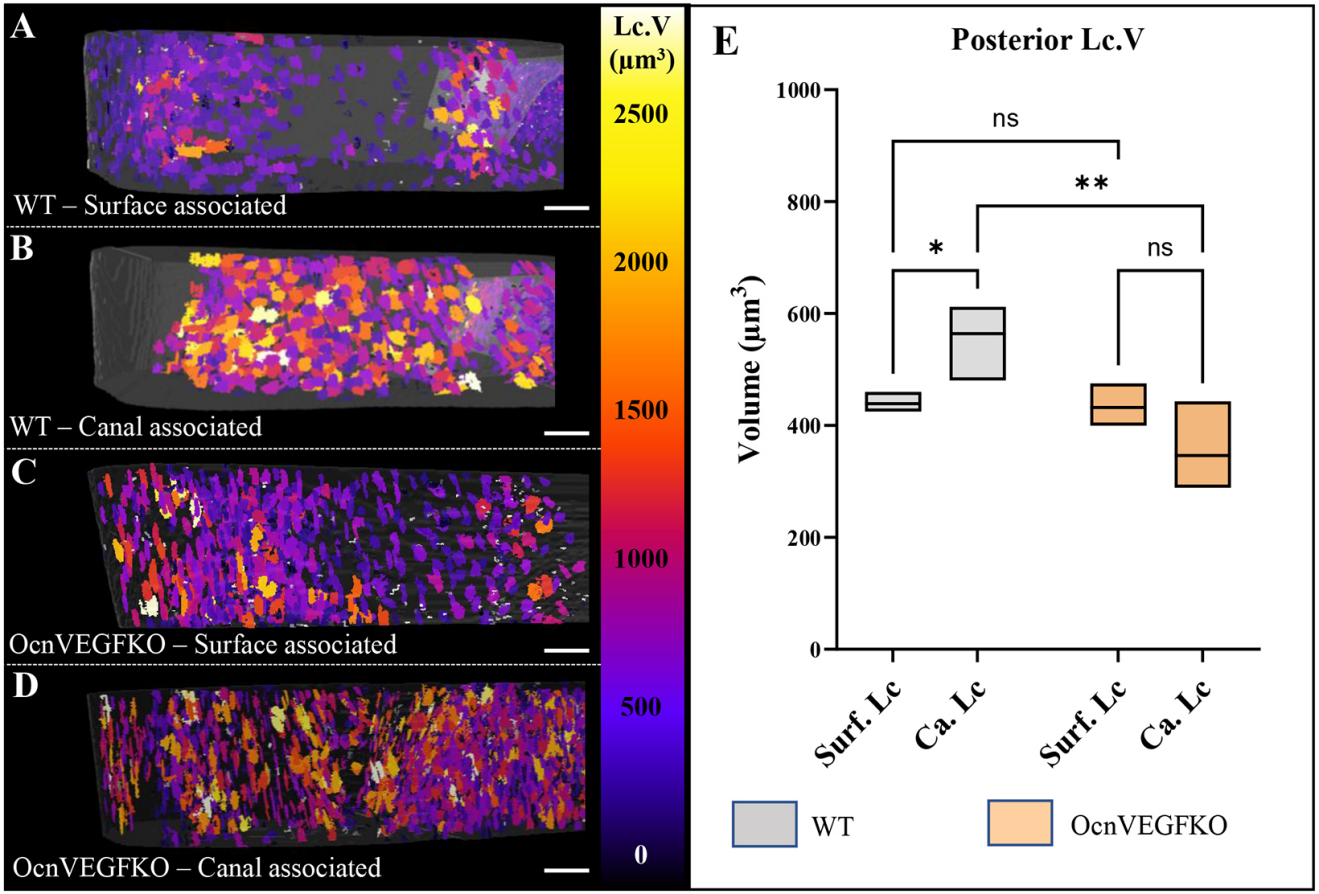
**Conditional VEGF deletion leads to a homogeneous population of osteocyte lacunae in the posterior region.** (A-D) 3D visualisation of surface-associated (A,C) and canal-associated (B,D) osteocyte lacunae within the posterior TFJ of 16-week-old male WT and OcnVEGFKO mice. Large lacunae are shown in yellow and white, and small lacunae are shown in purple. (E) Quantification of average Lc.V in WT and OcnVEGFKO mice. Ca. Lc, canal-associated lacunae; Surf. Ca, surface-associated lacunae. Scale bars: 200 μm. Data shown as mean±s.d. and assessed by a two-way ANOVA with subsequent post-hoc analysis, *n*=3 bones. ns, not significant; *P<0.05, **P<0.01.

Regionalisation of bone cortices in line with our previous results revealed that, in WT TFJs (Fig. 7A,B), posterior canal-associated lacunae were larger (564±42 µm^3^) than surface-associated lacunae (439±11 µm^3^, *P*=0.034). This is in contrast to OcnVEGFKO TFJs (Fig. 7C,D), in which there were no significant differences in average volume between canal-associated lacunae (346±49 µm^3^) and surface-associated lacunae (432±22 µm^3^, *P*=0.120) (Fig. 7E). Of note, the volume of posterior canal-associated lacunae in WT mice was greater than that of the equivalent in OcnVEGFKO mice. Together, these results suggest that loss of VEGF signalling by osteocalcin-expressing cells can regulate the mean volume of canal-associated lacunae present and therefore that bone derived-VEGF signalling is modular, localising only to specific vascular compartments in the TFJ.

## DISCUSSION

Using a newly developed automated regionalisation tool for SR CT imaging and 3D distance mapping techniques, we have characterised the spatial organisation and phenotypes of osteocyte lacunae surrounding intracortical canals in 3D within the murine tibial cortex. Our findings demonstrate distinct spatial variation in osteocyte lacunar morphology across the TFJ, with the posterior region exhibiting the highest cortical porosity and canal density, and largest lacunae, localised to vascular canals. This spatial organisation was lost following bone-specific VEGF deletion, leading to homogeneity in porosity and loss of regional lacunar–vascular associations seen in WT bones. These results suggest that VEGF enables spatially coordinated osteocyte–vascular architecture and structural phenotypes within cortical bone. Although vascular heterogeneity in bone has previously been described (Chen et al., 2020), this is the first time that heterogeneous osteocyte lacunae associated with vascular canals have also been identified and is suggestive of reciprocal interactions.

Regionalisation across the cortex has here proven to be a useful tool, enabling heterogeneity in osteocyte lacunae volume to be revealed successfully in mice, which is consistent with previous findings in both human and ray-finned fish cortical bone, suggesting that variability in osteocyte morphology is an evolutionary conserved feature across taxa (Canè et al., 1982; Hannah et al., 2010; Carter et al., 2013; Davesne et al., 2020). This morphological diversity has been proposed to reflect adaptive responses to local mechanical and metabolic environments, ultimately contributing to cortical bone integrity and ability to resist and adapt to bone-specific strain (Hunter and Agnew, 2016).

Skeletal pathological conditions further highlight the functional relevance of this heterogeneity; osteopenic bone is characterised by enlarged osteocyte lacunae, whereas osteoarthritic bone presents smaller lacunae (van Hove et al., 2009). Similarly, in human bone, microvascular disease has been associated with larger osteocyte lacunae, suggesting a relationship between vascular health and osteocyte morphology (Zanner et al., 2023). The functional implications of this lacunar heterogeneity remain an area of active investigation, with mechanotransduction and pericanalicular remodelling emerging as key mechanistic hypotheses (Tiede-Lewis and Dallas, 2019).

Although the potential for a heterogeneous distribution of large osteocyte lacunae playing a role in the adaptation to reduced canalicular connectivity requires further examination, the reporting of heterogeneity in osteocyte canalicular length and load-induced fluid flow at murine TFJs (van Tol et al., 2020) suggests that these adaptions to LCN degeneration are region specific. Alternatively, the role of osteocytes in bone remodelling (Kennedy et al., 2014; Bonewald, 2011) could suggest that the heterogeneous distribution of large osteocyte lacunae facilitates regional bone formation – such as during perilacunar remodelling. Regulation of osteocyte lacunae and canalicular volume through local matrix resorption/deposition is observed in response to lactation (Qing et al., 2012), various stimuli (Canalis et al., 2013) and glucocorticoid treatment (Lane et al., 2006).

Enlargement of osteocyte lacunae has been reported during lactation seen by Qing et al. (2012) and is suggestive of a tightly regulated and potentially reversible process driven by hormonal cues. A caveat to our current study is the lack of females as a comparison, and better understanding of the involvement of gonadal hormones in lacunar: vascular arrangement is important given sexual dimorphism of the bone vasculature and the prevalence of osteoporosis in women post menopause. During lactation, the authors observed expression of resorptive markers in osteocytes located both near bone surfaces and deep within cortical bone, indicating that this remodelling response is not uniformly distributed across the osteocyte network (Nakano et al., 2004; Powell et al., 2011). Therefore, the population of large lacunae at the posterior TFJ region could potentially be lacunae that had previously or are currently resorbing the surrounding matrix, increasing their lacunar volume. The absence of a similar response in nonloaded calvarial bone implies that mechanical cues modulate the hormonal response, contributing further to the heterogeneity seen (Wronski and Morey, 1982). Additionally, the impaired lacunar remodelling observed by Qing et al. (2012) in PTH1R conditional knockout mice during lactation confirms the essential role of PTHR1 signalling in mediating these localised changes in osteocyte size. These findings show that the distribution and size of osteocyte lacunae are dynamic and hormonally regulated in a regionally heterogeneous manner (Kovacs, 2005; O'Brien et al., 2008). Should large lacunae differentially affect the composition of the local matrix compared to small lacunae in specific regions of bone, their potential for the regulation of bone mineral may provide avenues for the research of region-specific bone anabolic agents.

Previous studies have hypothesised that variation in morphology of the osteocyte lacunae can result in the mechanoresponse of osteocytes being modified (Hemmatian et al., 2018, 2021). Alterations in LCN volume can inhibit bone mechanosensitivity; increased LCN volume and reduced connectivity in aged mice dampened the mechanosensory response. Osteocytes in these mice bones could be editing local strain environments by changing the shape of their lacunae by perilacunar remodelling, allowing them to maintain biological responses (Hemmatian et al., 2021). Expansion of the osteocyte lacunae may rescue this deficit, suggesting that enlargement of the pericellular space could compensate for reduced LCN connectivity (Schurman et al., 2021). Intriguingly, the posterior region of the TFJ highlighted here experiences low levels of compressive and tensile stress during loading in comparison to other regions, suggesting that mechanical strain could negatively regulate the presence of intracortical canals. Thus, we suggest that the absence of strain could permit the development of intracortical canals, which at high-strain regions could reduce bone strength, leading to fracture. This hypothesis could also provide an explanation for the observation that large lacunae appear to surround posterior intracortical canals, with low-strain regions being potentially more able to sustain large lacunae due to their reduced mechanical load, versus those located at the periosteal and endosteal surfaces. Although high-resolution CT studies of lacunae are commonly used as a proxy for osteocytes, this has not yet been robustly validated in mice. In humans, however, Qiu et al. (2002) demonstrated that 97% of variation in osteocyte density mapped to lacunar density in human trabecular bone taken from transiliac biopsies, and Bloch et al. (2012) reported that only 14% of lacunae were empty by age 80 years in the rib cortex. Further, early studies from Power et al. (2002) predicted that, in people aged 70-90 years, osteocyte lacunar occupancy is statistically associated with bone turnover, implying that high turnover (e.g. young bone) might favour lacunar occupancy. Further work validating osteocyte occupancy in mice, e.g. with age, is required to most effectively interpret our data on heterogeneity in lacunae density and size and to link directly to osteocyte function.

Our findings that intracortical canal distribution is heterogeneous is not specific to the murine TFJ: at the murine femoral midshaft, the lateral region possesses more canals than the posterior region (Schneider et al., 2007), whereas in humans (Perilli et al., 2015), the medial proximal femoral shaft houses more intracortical canals than the anterior and lateral regions. This suggests that the abundance of intracortical vasculature may vary between anatomical sites; however, the mechanism underpinning vascular canal heterogeneity described in these studies remains unspecified.

We have previously reported that the posterior region of the TFJ exhibits the largest cortical thickness across all regions and that a higher vascular canal density will be critical for effective vascular perfusion as well as osteocyte survival (Goring et al., 2019), and, as mentioned, a proportion of these osteocytes will be positioned at distances greater than 100 µm from any other bone surfaces and vascular supply. The unique ‘reliance’ of this population of osteocytes to the intracortical canals in the posterior region may have evolved and compartmentalised to optimise and quicken processes such as oxygen and growth factor diffusion from the cortical canals to the more isolated osteocytes deeply buried within the bone. Upon reflection, it is therefore possible that the vasculature and osteocytes within lacunae utilise distinct and reciprocal signalling mechanisms in the posterior region to enable this unique relationship to exist most effectively. Variability in local growth factor production or sensitivity across the regions’ TFJ may help to inform this hypothesis; therefore, we finally sought to study the structural phenotypes previously described in the absence of bone-derived VEGF. Here, we found that the unique vascular: lacunar arrangement characterised at the posterior TFJ in male mice was lost in the absence of VEGF, with porosity being homogeneously distributed and contributing to the whole-bone pathological poorly mineralised phenotype described (Goring et al., 2019). This suggests that VEGF is required to maintain vascular and lacunae arrangements across all compartments of the TFJ. This finding advances our knowledge of the angiogenic, sexually dimorphic effects of VEGF (Goring et al., 2019) and its regulation of the bone microstructure deficits as well as increased sclerostin expression (an inhibitor of osteoblast function and osteoid formation), suggesting that loss of VEGF affects osteocyte function and organisation either directly or indirectly through effects on the canals themselves.

Moving forward, detailed assessment of the mineral surrounding canal-associated osteocytes in health, disease and treatment states could also provide insight to potentially unique functionality of these canal-associated osteocytes. Currently, techniques that enable the 3D assessment of osteocytes within their lacunae and subsequent relation to the mineral matrix (i.e. without decalcification) are limited, with the majority of applications lacking sufficient soft-tissue contrast and resolution to visualise the osteocytes within lacunae. Furthermore, the recently described VEGF-regulated lymphatic system within bone and its roles in bone regeneration provide yet another avenue for intra-cell-population communication in response to mechanical or biological stimuli (Biswas et al., 2023).

In summary, our findings highlight a region-specific coupling between osteocyte lacunae size and vascular canal positions within the cortical bone, and regulation by bone-derived VEGF. The presence of enlarged osteocyte lacunae in vascular-rich, low-strain regions such as the posterior TFJ suggests that local osteocyte–vascular interactions will contribute to the spatial control of bone microarchitecture and mineralisation. Disruption of this coupling in VEGF-deficient bone, alongside increased cortical porosity and mineralisation deficits, described in our previous work (Goring et al., 2019), defines the importance of this regulatory circuit in maintaining cortical bone integrity spatially. Our findings have broad implications for understanding bone fragility in conditions such as osteoporosis, in which altered vascularisation and impaired osteocyte function may contribute to regional vulnerability and fracture risk.

## MATERIALS AND METHODS

### Animals

Male C57BL/6 WT mice were bred in house and maintained until 13 months of age. Post mortem, right tibiae were dissected and fixed in 4% paraformaldehyde (pH 7.4 in PBS) for 48 h on rotation (Núñez et al., 2018) and stored in 70% ethanol. Second-generation OcnVEGFKO mice were also used and bred as described (Goring et al., 2019). Right tibiae were collected from 4-month-old male OcnVEGFKO mice and WT (VEGF flx/flx) littermates and prepared as described (*n*=3). Animal studies were conducted in accordance with the UK Animals (Scientific Procedures) Act 1986 and approved by the University of Southampton Animal Welfare and Ethical Review Body and complied with Animal Research: Reporting of *In Vivo* Experiments guidelines.

### SR CT

SR CT was undertaken at Diamond Light Source (Harwell, UK) at Beamline I13-2 (Proposal 21843). In preparation for scanning, right tibiae were dehydrated and mounted in paraffin wax to prevent sample movement during scanning. The TFJ was selected as the field of view (FOV) (1.8×1.2 mm^2^), and, for each scan, 2000 projection images were acquired over a range of 360°. A photon energy of 18.5 keV was used with an exposure time of 500 ms. Images were acquired using the pco.4000 camera and imaged at 10× magnification to provide an isotropic voxel size of 1.65 µm. Datasets were corrected for ring artefacts and reconstructed using standard filtered back projection. The FOV was selected to incorporate a small region above the TFJ, with the first point of continuous contact between the tibia and fibula defined as the beginning of the TFJ. 500 slices were selected from this site of contact and used for analysis. The TFJ was set as the region of interest (ROI) to provide consistency in the analysed regions; further, the TFJ is an anatomical site consisting solely of cortical bone.

### Alignment of the TFJ

From each scan, 500 slices of 8-bit greyscale image stacks starting at the TFJ were selected and imported into Fiji (Schindelin et al., 2012). Global iterative thresholding was used to convert greyscale images into binarised datasets, permitting separation of the bone cortex from soft tissue and air (Leung and Lam, 1996). To standardise region separation across murine tibial samples, the BoneJ moment of inertia function (Doube et al., 2010) was utilised to align the bone scan's principle axis to the image stack's *x*-, *y*- and *z*-axes, after which images were rotated −45° and canvas size was adjusted to produce equal height to width ratio, e.g. 4000×4000 pixels.

### Extraction of cortical porosity and pore quantification

Aligned binary TFJ image stacks were duplicated, and intracortical pores were filled with a series of binary dilate functions. Upon complete closure of intracortical pores, the cortical mask was dilated to return the mask to the size of the original cortex. The cortical mask was inverted and combined with the thresholded stack through image addition, yielding cortical porosity as described (Núñez et al., 2018; Schneider et al., 2007). BoneJ's particle analyser (Doube et al., 2010) was utilised to sort cortical porosity into noise (0-25 µm^3^), osteocyte lacunae (25-2500 µm^3^) and intracortical canals (>2500 µm^3^), as previously described (Núñez et al., 2018; Goring et al., 2019). Cortical porosity [Ct.Po (%)], canal number density [N.Ca/Ct.TV (mm^−3^)], lacuna number density [N.Lc/Ct.TV (mm^−3^)], canal volume density [Ca.V/Ct.TV (%)], lacunar volume density [Lc.V/Ct.TV (%)], average canal volume [Ca.V (μm^3^)] and average lacunar volume [Lc.V (μm^3^)] were calculated in concordance with standard nomenclature for bone morphometry (Bouxsein et al., 2010). Lacunar volume distributions were visualised in Dragonfly. Osteocyte lacunae containing image stacks were imported and converted into multi-ROI datasets so that each osteocyte lacunae was treated as its own ROI. Object analysis tool was then used to quantify lacunar volume and mapped using the ‘warm metal’ look-up table (LUT).

### Automated regionalisation of cortical porosity at the TFJ

Following TFJ alignment and isolation of the cortical porosity, quadrants were identified via selection of coordinates within Fiji. For a canvas size of 4000×4000 pixels, quadrants of 2000×2000 pixels were selected using ‘SPECIFY’ function. ‘MAKE INVERSE’ was used to select the remaining three quadrants, and ‘FILL’ was used to fill these three quadrants with pixels with an intensity of 0 (black pixels, denoting empty space), leaving one quadrant with our pixels of interest (attributable to bone or cortical porosity). Specifying the upper left quadrant (0-2000 pixels in *x*, 0-2000 pixels in *y*) isolated the posterior, the upper-right quadrant (2000-4000 pixels in *x*, 0-2000 pixels in *y*) isolated the lateral, the lower-left quadrant (0-2000 pixels in *x*, 2000-4000 pixels in *y*) isolated the medial, and the lower-right quadrant (2000-4000 pixels in *x*, 2000-4000 pixels in *y*) isolated the anterior, region of the TFJ.

Cortical total bone volume (Ct.TV) was calculated with BoneJ's particle analyser for each whole TFJ as well as each region. 3D visualisation of regional bone masks and regional cortical porosity was performed using Dragonfly 2020.2 [Object Research Systems (ORS)]. This segmentation technique allocated 28.20% (±1.09%) of the total cortical volume to the anterior region, 35.27% (±1.49%) to the posterior region, 16.67% (±0.23%) to the lateral region and 19.85% (±2.37%) to the medial region. Image processing workflows of the extraction of cortical porosity alignment of bone cortices for regionalisation is illustrated in Fig. 1A,B.

### 3D lacunar distance mapping

Once osteocyte lacunae and intracortical canals were isolated, an image subtraction (binary cortical mask minus binary intracortical canal image stacks) was used to create a cortical mask perforated by intracortical canals (workflow included in Fig. 2A). The endosteal bone surface, periosteal bone surface and intracortical canals were designated as the sites of potential vascular supply (Fig. 2C).

Images are then inverted to convert the vascular surfaces to the ROI and imported into Dragonfly. A distance map was then created from the inverted intracortical canal mask to map the distance of each pixel within the bone cortex to the nearest surface of the inverted intracortical canal mask. Each pixel was then attributed a pixel intensity that directly relates to the distance from a vascular surface, in steps of the image resolution. A bone surface was attributed with a distance of ‘0’, so received a pixel intensity of 0. Next, lacunae image stacks were imported into Dragonfly and converted to a multi-ROI so that every lacunae was treated as a separate entity. Each lacuna was superimposed onto the distance map, yielding a pixel intensity reflective of the pixels within that lacuna's distance from a bone surface. The smallest of these distances was then isolated to yield ‘minimum lacunar distance’ and coloured using the ‘temperature’ LUT. The minimum lacunar distance reflected the shortest distance between the lacunae and a potential vascular supply. This was completed for each lacunae and averaged to yield the mean minimum lacunar distance (Mm.Lc.D).

In addition to Mm.Lc.D, lacunae were binned based on their proximity to a bone surface – those within 25 µm, 50 µm, 75 µm and >100 µm. The percentage of lacunae attributable to each of distance bins was then calculated and termed lacunar percentage distance distribution (%Lc.D.D). 100 µm was selected as the upper boundary for lacunar distance, as the literature has reported that this is the maximum distance that an osteocyte can survive while relying solely on diffusion (Zahm et al., 2010).

To determine the contribution of vasculature of the bone cortex, including bone surfaces and intracortical canals, in the support of lacunae, the inverted cortical mask was imported into Dragonfly, and a distance map was calculated between exclusively the endosteal and periosteal bone surfaces, in the absence of intracortical canals (canal-excluded distance mapping, termed ‘Ca.Excl’) (Fig. 2B). This was then compared to the lacunar distance mapping analysis including intracortical canals (canal-included distance mapping, termed ‘Ca.Incl’) (Fig. 2C). Should the minimum distance between and osteocyte lacunae and a potential vascular supply remain the same in both Ca.Incl and Ca.Excl analysis, then a bone surface is the nearest vascular supply and a lacuna was termed surface associated. Should canal inclusion alter the minimum lacunar distance, then intracortical canals reduce the distance between that osteocyte lacuna and a potential vascular supply, and this lacuna was classed as canal associated.

An ‘association filter’ was applied to each lacuna in each region, sorting lacunae as canal associated and surface associated based on whether individual lacunae are closest to an intracortical canal or a bone surface. From this, the percentage of lacunae associated with intracortical canals was calculated within each region (Fig. 2). Should the minimum distance between each osteocyte lacunae and potential vascular supply remain the same in both Ca.Incl and Ca.Excl analysis, the bone surface was allocated as the nearest vascular supply.

### Statistical analysis

To investigate the role of regionalisation in a bone's microstructural characteristics, a one-way ANOVA was used with Tukey's multiple comparisons to assess statistical differences between groups as detailed in figure legends. For the assessment of cortical microstructural parameters in OcnVEGFKO versus WT littermate controls, a paired two-way ANOVA was employed to compare littermates. Values are shown as mean±s.d., and *P*<0.05 was deemed statistically significant. All statistical tests were performed in GraphPad Prism 6.0.
